# Tunable Electronic Transport of New-Type 2D Iodine Materials Affected by the Doping of Metal Elements

**DOI:** 10.3390/molecules28207159

**Published:** 2023-10-19

**Authors:** Jie Li, Yuchen Zhou, Kun Liu, Yifan Wang, Hui Li, Artem Okulov

**Affiliations:** 1 School of Materials Science and Engineering, Jiangsu University of Science and Technology, Zhenjiang 212100, China; zhouyuchen23@163.com (Y.Z.); liu_kun@163.com (K.L.); 18001759763@163.com (Y.W.); 2 Key Laboratory for Liquid-Solid Structural Evolution and Processing of Materials, Ministry of Education, Shandong University, Jinan 250061, China; 3 M.N. Mikheev Institute of Metal Physics, Ural Branch of Russian Academy of Sciences, Ekaterinburg 620077, Russia; okulovartem@imp.uran.ru

**Keywords:** new-type 2D iodine materials, electron transport, doping of metal elements, applying the bias, first principles

## Abstract

2D iodine structures under high pressures are more attractive and valuable due to their special structures and excellent properties. Here, electronic transport properties of such 2D iodine structures are theoretically studied by considering the influence of the metal-element doping. In equilibrium, metal elements in Group 1 can enhance the conductance dramatically and show a better enhancement effect. Around the Fermi level, the transmission probability exceeds 1 and can be improved by the metal-element doping for all devices. In particular, the device density of states explains well the distinctions between transmission coefficients originating from different doping methods. Contrary to the “big” site doping, the “small” site doping changes transmission eigenstates greatly, with pronounced electronic states around doped atoms. In non-equilibrium, the conductance of all devices is almost weaker than the equilibrium conductance, decreasing at low voltages and fluctuating at high voltages with various amplitudes. Under biases, K-big doping shows the optimal enhancement effect, and Mg-small doping exhibits the most effective attenuation effect on conductance. Contrastingly, the currents of all devices increase with bias linearly. The metal-element doping can boost current at low biases and weaken current at high voltages. These findings contribute much to understanding the effects of defects on electronic properties and provide solid support for the application of new-type 2D iodine materials in controllable electronics and sensors.

## 1. Introduction

Currently, one-atom-thick 2D nanomaterials are catching researchers’ attention because of their fascinating structures, outstanding properties and promising applications [1,2]. The elemental 2D materials are surely striking among the 2D family due to their simples chemical composition and amazing characteristics [3,4]. For example, the most typical one is graphene (group-IVA), with high electron mobility, Young’s modulus, and thermal conductivity [5,6]. Unlike the honeycomb structure based on six-membered carbon rings of graphene, silicene, germanene, and stanene (group-IVA) possess a buckled hexagonal honeycomb lattice with variable electronic structures [7,8]. Borophene (group-IIIA) shows metallic features and highly anisotropic electronic properties [9]. Unlike the above, black phosphorene (group-VA) is a semiconductor with a tunable band gap and shows anisotropic properties on the surface [10]. In recent years, arsenene [11], anitmonene [12], and bismuthene [13] (group-VA), as well as selinene [14] and tellurene [15] (group-VIA), have been reported theoretically and experimentally. Elemental 2D nanomaterials have shown great potential in electronics [16], sensing [17], energy storage [18], photothermal therapy [19], and other applications [20].

Noticeably, elemental 2D nanomaterials in the group-VIIA have begun to receive attention. Two-dimensional binary iodides, including BiI_3_ [21], PbI_2_ [22], and RhI_3_ [23], have been proved to behave excellent performance in photonics. Like these binary iodides, iodine is a layered semiconductor. Atom-thin 2D iodine materials have now been successfully prepared via liquid phase stripping [24]. Such 2D iodine overcomes the deficiencies of slow battery kinetics, poor rate capacity, low conductivity, etc., from the crystal, showing great potential for use in high-performance rechargeable batteries [25]. It should be noted that iodine is a molecular system in which iodine molecules are connected to the layer plane via halogen bonds rather than covalent bonds, making it a fundamental point of interest [26]. Therefore, the special structure of 2D iodine material combined with the nanometer size effect shows great application value in terms of high-pressure physics, the energy field, the optoelectronics field, etc. [27,28,29,30,31]. Regardless, 2D iodine-based research on emerging materials is in its infancy and still requires a great deal of research.

High pressure can effectively regulate the physical and chemical properties of materials [32,33,34]. The iodine molecule, as one of the seven homonuclear diatomic molecules, shows stability under ambient conditions and is high-profile as a result of successive transitions (a superconductive state [35] or metallization [36]) induced by the pressure. At ambient pressure, solid iodine is an orthorhombic molecular crystal (space group, Cmca; phase I). It has been demonstrated that there are no signs of structural phase transition when the pressure is below 23.2 GPa [37]. The conductance strengthens by eight orders of magnitude with the pressure to 12 GPa [36]. When the pressure is high enough, iodine molecules are close to each other and cause the conduction and valence bands to overlap, and as a result, iodine is converted into molecular metals. Pressure-induced photon-generated carriers increase rapidly, leading to the increase in the photoelectric properties of iodine [38]. The special structure and excellent properties caused by high pressure make the 2D iodine structure more attractive and valuable for use under high pressures.

However, there has not been nearly enough research performed on 2D iodine structures under high pressure, as well as their electronic transport properties and applications. Here, a 2D iodine monolayer under a certain high pressure is the research subject. We theoretically study the influence of metal-element doping on the equilibrium and non-equilibrium electron transport properties, including the conductance, electron transmission, and current–voltage characteristics. Electronic structures and charge transferring states are also well explained.

## 2. Results and Discussion

**Figure 1:**
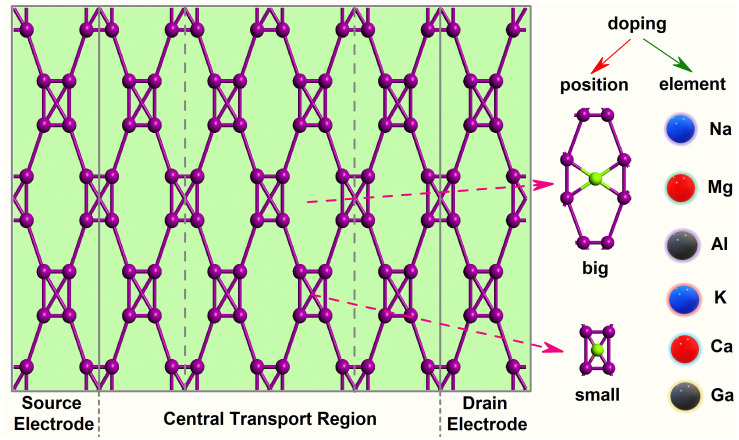
Structural illustration of the devices based on no-doped and doped iodine monolayers.

Here, we studied a new-type 2D iodine structure, which is under a high pressure of 15 GPa (see Figure 1). Just as Li et al. reported, the pressure would make iodine atoms more clustered together, so that 2D iodine shows much shorter atomic distances of less than 3 Å and no band gap under the pressure of 15 GPa, different to the general condition [39]. However, iodine is a 2D layered molecular crystal with covalent intramolecular bonds, so atoms are regularly bonded in pairs. Here, four iodine atoms form a rectangle, where each iodine atom is bonded to one iodine atom in another rectangle. As a result, iodine atoms from four rectangles form an eight-membered ring. Significant electronic interactions occur with the next-nearest neighbors of each atom. Taking into account the difficulty of the bonding, the doped metallic elements are selected from groups 1, 2, and 3, which are Na, K, Mg, Ca, Al, and Ga, respectively. There are two types of doping sites for 2D iodine depending to its structural characteristics. As Figure 1 shows, one is in the big 8-I ring, and the other is in the small 4-I ring.

To study the electrical sensitivity to metal atoms, electronic devices are constructed in Virtual NanoLab, as shown in Figure 1. The device actually has a two-probe configuration. For the left and right electrode regions, the system should be periodic along the transport direction (the C direction). Besides the transferring material, the central region includes the left and right electrode extensions in order to screen out the perturbations from the scatterer and solve a bulk problem for the fully periodic electrode cell. Due to their being no band gap [39], the material itself is used as the electrode. Simulated electronic devices are within a supercell with over 15 Å of vacuum space to allow electrostatic interactions to decay for the system [40]. The device with the original new-type 2D iodine is denoted as Device no-doped. The doped 2D iodine-based devices are written as Device Na-big, Device Na-small, Device Mg-big, Device Mg-small, Device Al-big, Device Al-small, Device K-big, Device K-small, Device Ca-big, Device Ca-small, Device Ga-big and Device Ga-small, respectively, according to the type and sites (big 8-I ring and small 4-I ring) of the doped element.

### 2.1. Equilibrium Electron Transport

**Figure 2:**
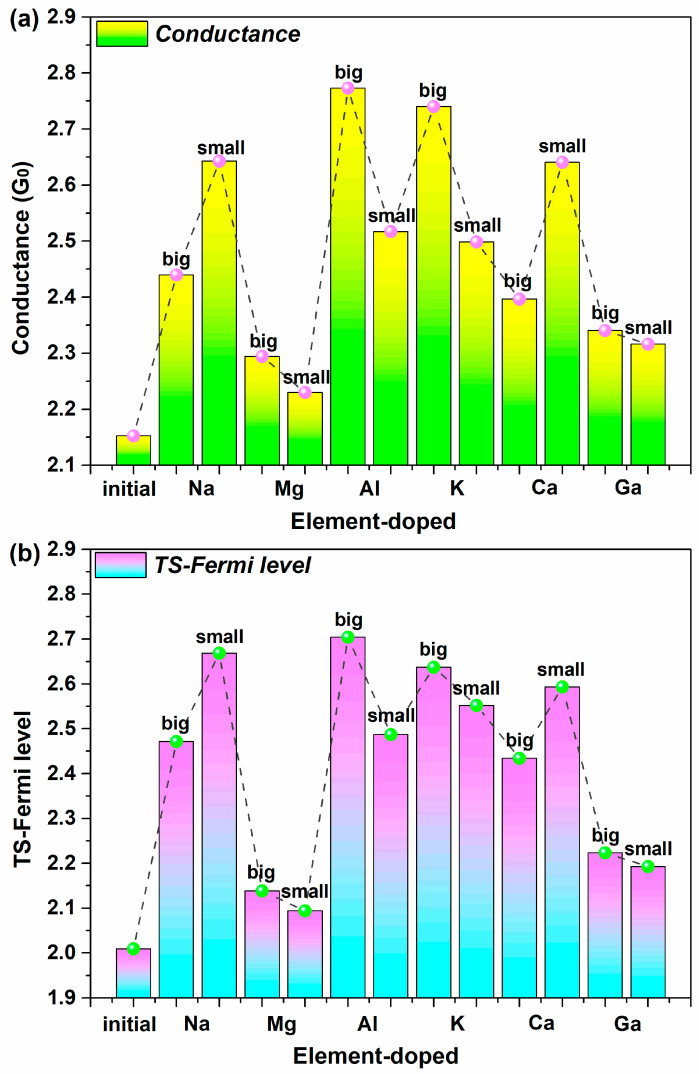
(**a**,**b**) Comparisons between equilibrium quantum conductance and electron transmission at the Fermi level among the undoped and doped iodine-based devices.

Firstly, we study the sensitivity of equilibrium conductance to metal elements. Figure 2a shows the comparison between equilibrium conductance among all new-type 2D iodine-based devices before and after doping. Obviously, the conductance of doped devices is much stronger than the original device. The doping position has a different influence on conductance for different doped metal elements. The devices doped in “big” site (called “big” doped devices) exhibit stronger conductance than the devices doped in “small” site (called “small” doped devices) with the elements Al and K. Moreover, the doping with these two elements in medium atomic numbers contributes to the most pronounced enhancement in conductance. The “small” doped devices show higher conductance values than the “big” doped devices for the devices with the doped metal elements Na and Ca in larger atomic numbers, and the influence of these two elements is also strong on the conductance value, though not as obviously as Al and K. Markedly different from the above, the doping site exhibits little effect on conductance for Mg-doped devices and Ga-doped devices, and the two types of elemental doping also show the least noticeable increases in conductance. Overall, both of the elements in Group 1 can enhance the conductance dramatically and show better doping effects than the four other elements in groups 2 and 3, which present significant influencing differences within the same main group.

To investigate the differences in conductance, Figure 2b shows the transmission coefficient of all the devices at the Fermi level (E_F_), which expresses the transport capacity of electronic devices [41]. In contrast to the original device, doped devices have a significantly strong electron transmission. On the whole, most doped metal elements can dramatically boost the electron transmission, and there is a small difference between the transmission coefficients of doped devices. The doping site also exhibits varying influences on the electron transmission for various doped metal elements, which is consistent with the conductance, but this difference in impact is not as obvious as that on the conductance. 

In detail, for the devices with the doped elements Al and K, the “big” site shows a greater advantage than the “small” site regarding the improvement in the electron transmission. When doped with metal elements Na and Ca, “small” doped devices tend to have higher electron transmission values than “large” doped ones. Unlike the above, the Mg-doped device exhibits the close value in the “big” and “small” sites, and the same is true for the Ca-doped device. Furthermore, these two elements demonstrate the minimum increase in the electron transmission coefficient. In general, both elements of Group 1 have a significant increase in electron transmission and show a superior doping effect compared to the other four elements of groups 2 and 3, which exhibit extreme electron transmission values. Thus, the electron transmission at E_F_ can well explain the conductance in the equilibrium for these new-type 2D iodine-based devices.

**Figure 3:**
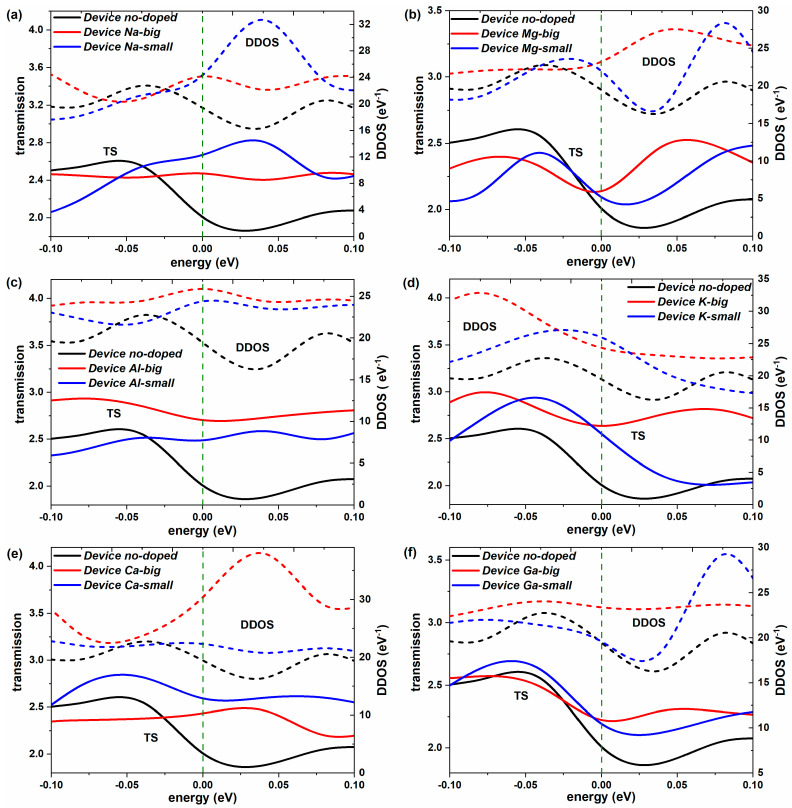
(**a**–**f**) Equilibrium electron transmission and device density of states (DDOS) of the comparison between Device no-doped and the iodine devices doped with Na, Mg, Al, K, Ca, and Ga, respectively.

Further, the equilibrium transmission spectrum in [−1 eV, 1 eV] is given and plotted in solid lines in Figure 3. Remarkably, the K-doped devices present much stronger electron transmission than the Device no-doped in the whole energy range. The electron transmission coefficients of Device Al-big and Device Ca-small are also obviously larger than Device no-doped, and Device Ga-small shows slightly stronger electron transmission than Device no-doped near E_F_, which fully demonstrates the advantage of the doping position. However, compared to Device no-doped, other types of doping devices present weaker electron transmissions in a quite small part of [−1 eV, 0 eV] while showing notably greater coefficients in the rest of the energy interval. It signifies that metal-element doping can improve the entire transmission spectrum around E_F_. For the role of the doping position, the “big” site is superior to the “small” site for Al-doped and K-doped devices, while the “small” site has an advantage over the “big” site for Na-doped and Ca-doped devices, which is consistent with the effect on the conductance and electron transmission at E_F_. For the special Mg-doped and Ga-doped devices, the influence of the doping position is uncertain, reflected in the variational contrast relationships of transmission values at different energy intervals around E_F_. Moreover, it can be seen that the transmission probability around E_F_ exceeds 1 for new-type 2D iodine-based devices because more than one obviously strong electron transmission channel contributes to electron transport near E_F_ for such new devices [40].

The electronic structures of the devices are calculated to analyze the variations in the transmission spectrum resulting from the doping [42]. Figure 3 displays the device density of states (DDOS) of all devices in dashed lines. It can be observed that the shapes of DDOS–energy curves are pretty similar to those of the transmission (TS)–energy curves for all devices, except the Ca-doped and Ga-doped devices with tiny differences. It indicates that the DDOS is the determining inner factor of electron transport for the new-type 2D iodine-based devices [43]. The relative size relationships of DDOS values among Device no-doped, Device Na-big, and Device Na-small are consistent with those of the transmission coefficients in most of the energy range, ignoring very small negative energy intervals. The same is true with the Mg-doped, Al-doped, and K-doped devices. However, some anomalies appear in devices based on the doped elements of Ca and Ga with higher atomic numbers. Smaller DDOS brings about a higher transmission value, which mainly occurs in the comparison between two different doping positions. On the whole, the DDOS can well explain the differences between the electron transmission probability resulting from the doping and different doping sites.

In order to more intuitively describe the changes in the equilibrium electron transport characteristics, Table 1 presents the variations in and variation rates of all doped devices in terms of the equilibrium conductance, electron transmission possibility at E_F_, and the device density of states at E_F_. Clearly, in the equilibrium condition, the most noticeable change in conductance occurs for Device Al-big, followed by Device K-big and Device Na-small, compared to the undoped device. The devices doped with Mg show the smallest changes in conductance. The changes in electron transmission possibility are basically consistent with those of conductance among the various doped devices. So, Device Al-big, Device K-big, and Device Na-small exhibit a significant advantage in terms of enhancing electron transport capacity, which also possess stronger electron transport. In contrast, the doping with Mg goes against such enhancement, which also shows weaker electron transport.

In contrast, Device Ca-big displays the biggest change in DDOS, which does not show strong electron transport capacity. What’s more, a remarkable improvement in the DDOS can be found in the devices doped with K and Al. However, the three devices above, with excellent electron transport and corresponding enhancement effects, also exhibit such improvements in DDOS. Moreover, the doping with Mg and Ga brings on slight changes in DDOS, especially for Device Ga-small.

**Figure 4:**
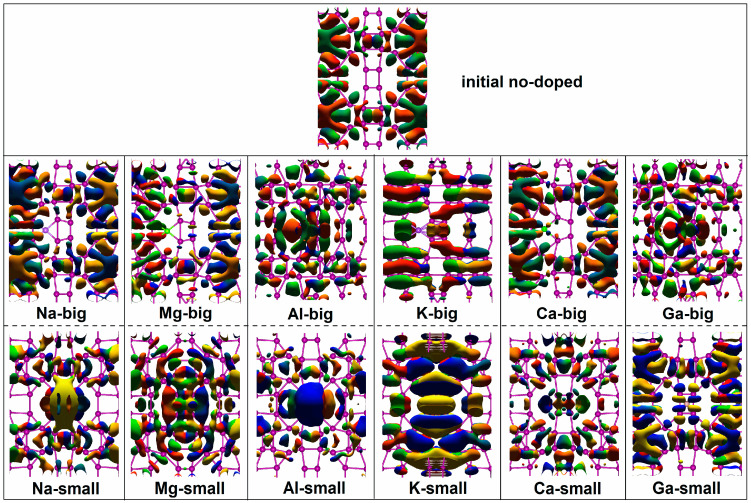
Transmission eigenstates of the undoped iodine-based device and all doped iodine devices with the same isovalue.

In addition to the analysis of intrinsic electronic structures, the spatial distribution of charge transfer is investigated to study the electron transport of the new-type 2D iodine-based devices. The dominant eigenstate of the transmission matrix at E_F_ is calculated and displayed in Figure 4. The transmission eigenstate is a complex wave function, corresponding to a scattering state that comes from the left electrode and travels towards the right [44]. There are two continuous distributions of electronic states from the left electrode to the right electrode for Device no-doped, which qualitatively demonstrates that the transmission coefficient exceeds 1.

Intuitively, the transmission eigenstates are slightly changed by “big” site doping, apart from the elements in Group 3, which show noticeable electronic states around the doping positions. For the other four elements, the transmission eigenstates are quite similar to the undoped device for the three doped elements of Na, Mg, and Ca while influenced by the doped K, with more than two transferring channels found throughout the entire device, albeit without conspicuous electronic states around the doping site, similar to the impacts of the elements in Group 3, which contribute to the stronger electron transport capacity of Device K-big. As a whole, for the “big” doping, the distributions of transmission states are seemingly affected by the elements in the same main group.

Unlike the above, the transmission eigenstate presents pronounced changes influenced by the “small” doping of metal elements. Apparent electronic states can be observed in the surrounding areas of the doped atom for all metal elements and are particularly strong for the four elements with larger atomic numbers. Around the doped atom, the morphology and distribution of electronic states vary with the doped element. Compared to the “big” doped device, the “small” doped one possesses more distributions of electronic states throughout the entire device, despite having weaker electronic states for the doped Na, internally causing stronger electron transmission of Device Na-small. The same applies to the Ca-doped devices. The Mg-doped devices show a similar number of transferring channels and strengths of transmission eigenstates on the two doping sites, and so does the Ga-doped devices, explaining the almost large transmission coefficients at the two doping positions. For Al-doped and K-doped devices, the “big” doping site promotes more transferring channels, stronger electronic states, and further stronger electron transmission compared to the “small” doping site.

### 2.2. Non-Equilibrium Electron Transport

**Figure 5:**
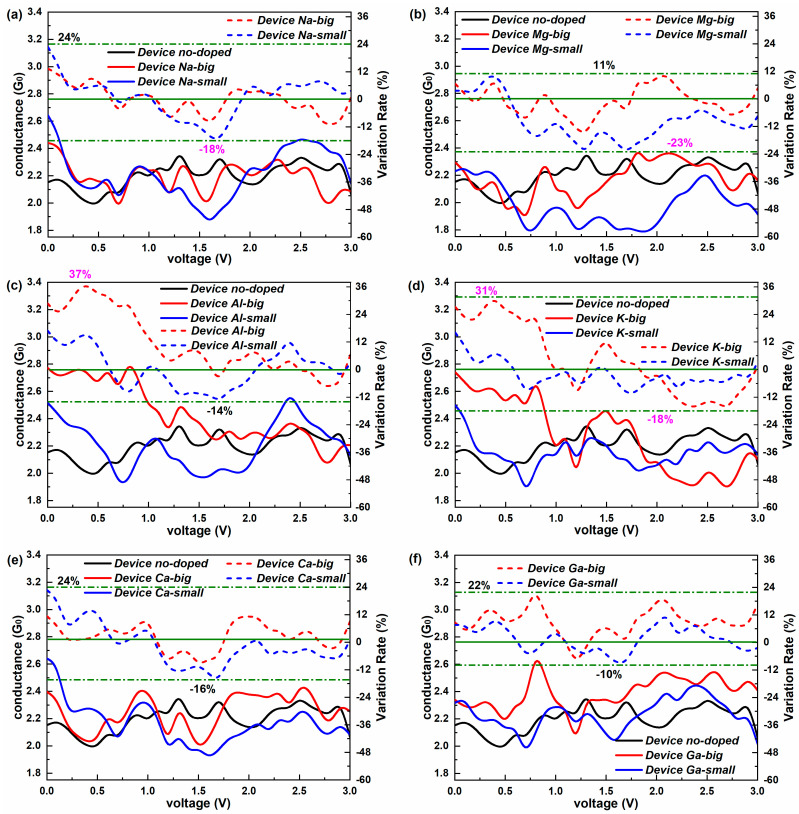
(**a**–**f**) Conductance–voltage curves of the undoped iodine-based device and the iodine devices doped with Na, Mg, Al, K, Ca, and Ga, respectively. The dashed lines show the variation rate of the conductance at various voltages for the corresponding doped devices relative to the undoped iodine-based device.

Non-equilibrium electron transport properties are also studied for the new-type 2D iodine-based devices. Figure 5 exhibits the conductance of all studied devices in the bias region of [0 V, 3 V]. For Device no-doped, a slight drop of conductance can be observed in [0 V, 0.5 V]. Then, the conductance grows gradually to the initial value and later keeps small fluctuations. So, the conductance value is less affected by the voltage for the no-doped device. It is clear that all devices have things in common: the conductance decreases under lower biases (less than about 0.6 V) and fluctuates as the bias increases. However, the amplitude of the variation differs. A small change in conductance (just 0.1 G_0_) can only be seen in the “big” doped devices with the elements from Group 3 in the initial descent stage. For the wave phase that follows, Device Na-big and Device K-small present the minimum changing range (about 0.3 G_0_), and both Ca-doping devices show relatively slight fluctuations in conductance with the bias, while the conductance alters the most (about 0.7 G_0_) with the change in the voltage for Device Al-big and Device K-big.

A significant difference of the conductance compared to the doping position can be found in the Mg-doped, Al-doped, and K-doped devices at some voltages. For the doping of the other types of metal elements, there is little difference in conductance between the “big” doping and “small” doping that is influenced by the bias. In addition, the conductance at all voltages is lower than the equilibrium conductance for the doped device for the elements in Group 1 and the “small” doping devices with the elements in Group 2. In contrast, the conductance at some voltages is a little higher than the equilibrium conductance for the doped device with the elements in Group 3 and the “big” doped devices with the elements in Group 2. Therefore, under the influence of the voltage, the conductance is almost reduced for the undoped and doped devices.

To measure the doping effect quantitatively, Figure 5 also illustrates the variation rate of conductance relative to the undoped condition for the doped devices. Here, the variation rate is defined as the ratio of the conductance difference between one doped device and undoped device relative to the conductance of the undoped device. Obviously, all doped devices present positive variation rates, indicating the promotion of the doping to the conductance at initial small voltages, and show widely negative rates, representing the weakening effect of the doping on the conductance at medium biases. When applied by the bias, the best doping for enhancing conductance is Device Al-big and Device K-big with strength rates of over 30% (up to 37%), mainly at low voltages. The most effective attenuation for the conductance appears in Device Mg-small, with a variation rate of −23%. The effect of the other doping methods is relatively weak.

**Figure 6:**
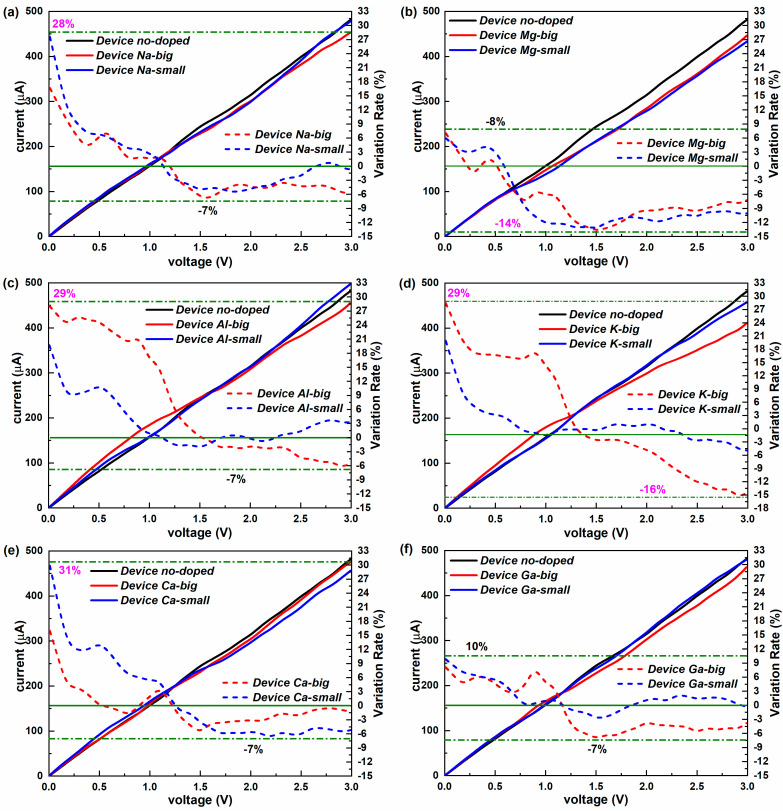
(**a**–**f**) Current–voltage curves of the undoped iodine-based device and the iodine devices doped with Na, Mg, Al, K, Ca, and Ga, respectively. The dashed lines show the variation rate of the current at various voltages for the corresponding doped devices relative to the undoped iodine-based device.

The current–voltage characteristics are one element of practical and important non-equilibrium performance [45]. Figure 6 presents the current–voltage curves of all devices, as well as the corresponding variation rates. Similar to the variation rate of the conductance, the variation rate of the current is the ratio of the difference in current between one doped device and one undoped device divided by the current of Device no-doped. Unlike the fluctuating trends of the conductance, the current increases linearly with the bias for the doped and undoped new-type 2D iodine-based device. It is not easy to find the differences in the current between the two doping positions or the doping effect from the comparisons of values alone, because the changing range of the current is too wide at the voltage range of [0 V, 3 V]. So, the following analysis is mainly based on the variation rate of the current.

It is not hard to find that the current of the new-type device can achieve growth at low voltages and reduction at high voltages via the doping of metal elements. The specific doping effects of different sites vary with the type of the element. For the doped devices with Na and Mg in smaller atomic numbers, the effect (strengthened or weakened) on the current of the “big” doped device synchronizes roughly with the “small” doped device, and there is a small difference between the two in terms of the value of the current. Though the Ga-doped devices also present a slight gap between the two, Device Ga-small possesses a negative variation rate, while Device Ga-big shows a positive one, which means that the effect on the current of the “big” doped device does not synchronize with the “small” doped device. In contrast, huge discrepancies in the variation rates can be observed between the “big” doped device and the “small” doped device for the other three kinds of doped devices, especially for the Al-doped and K-doped devices at lower biases. Like the doped device with Ga in Group 3, the out-of-sync phenomenon of the doping effect also exists in the Al-doped devices due to different doping positions. Such an obvious out-of-sync phenomenon only appears in the doped devices with metal elements in Group 3.

However, in contrast, with the equilibrium condition, the reinforcement effect on the current fades at voltages for the doped devices, but the reduction effect can be achieved by applying higher biases. The doping of most elements can reach excellent results, with a variation rate of about 30% in terms of the raising impact on the current except Mg and Ga, and the choice of the doping position depends on the element type. The variation rate of −7% is easily accessible to the doped devices by applying a voltage, and Device K-big, Device Mg-big, and Device Mg-small all present the best weakening effect on the current, with a rate of about −15%. So, clearly, the Mg-doped doping can weaken the current by applying a certain bias whatever the doping site. The optimum intensifying and dampening impact can both be possible with the K-doped doping by varying the voltage.

## 3. Computational Methods

All structures were optimized before calculating electron transport properties. All calculations based on the first-principles theory, combining density functional theory (DFT) [46] with non-equilibrium Green’s function (NEGF) [47], was implemented in the Atomistix Toolkit (ATK) package [48]. The exchange–correlation function was a generalized gradient approximation (GGA) of Perdew–Burke–Ernzerhof (PBE), while the double-zeta plus polarization (DZP) basis was chosen for all atoms. K-point sampling was set to 1 × 5 × 50, and the transport direction was along the C axis. The density mesh cut-off for the electrostatics potential was 75 Ha. The electron temperature was set as 300 K. Structural optimizations use the quasi-Newton method until all residual forces on each atom are smaller than 0.05 eV/Å. The convergence criterion for the total energy was 10^−5^ via the mixture of the Hamiltonian.

The conductance *G* could be expressed in terms of the transmission function within the Landauer–Büttiker formalism [49,50]:

(1) $$G=G_{0}\int_{-\infty }^{+\infty }dET\left(E,V\right)\left(-\frac{\partial f\left(E\right)}{\partial E}\right)$$

The current through a molecular junction was calculated using the Landauer–Büttiker equation [51]

(2) $$I=\frac{2e}{h}\int_{-\infty }^{+\infty }dET\left(E,V\right)\left(f_{1}\left(E\right)-f_{2}\left(E\right)\right)$$

where *G*_0_ = 2*e*^2^/*h* is the quantum unit of conductance, *h* is Planck’s constant, *e* is the electron charge, $f_{1,2}\left(E\right)$ are the Fermi functions of source and drain electrodes, and $T\left(E,V\right)$ is the quantum mechanical transmission probability of electrons. Thus, it can be given as depicted in [51]

(3) $$T\left(E,V\right)=tr\left[\Gamma_{L}\left(E,V\right)G^{R}\left(E,V\right)\Gamma_{R}\left(E,V\right)G^{A}\left(E,V\right)\right]$$

where *G^R^* and *G^A^* are the stunted and advanced Green functions of the conductor part, respectively, and $\Gamma_{L}$ and $\Gamma_{R}$ are the coupling functions to the left and right electrodes, respectively.

## 4. Conclusions

We have systematically studied the doping influence of metal elements on the equilibrium and non-equilibrium electron transport properties of the devices based on the new-type 2D iodine structure. Our results show that the equilibrium conductance of doped devices is much stronger than those of the undoped device, and the doping position has varying influence on the conductance depending on the element type. The elements in Group 1 can both enhance the conductance dramatically and show better doping effects than the other elements in groups 2 and 3, well explained based on the electron transmission at E_F_. The transmission probability around E_F_ exceeds 1 for the new devices due to their being more than one obviously strong electron transferring channel. The metal-element doping can improve the entire transmission spectrum around E_F_. Essentially, the device density of states (DDOS) is the determining inner factor in electron transport for these devices and can well illustrate the differences of transmission coefficients from doping and the doping site. For the spatial distribution of charge transferring, the transmission eigenstates are slightly changed by the “big” site doping for most elements, and they are a semblable for the elements in the same group. In contrast, the transmission eigenstates change dramatically when influenced by the “small” doping of metal elements, presenting pronounced electronic states in the surrounding of the doped atom. In non-equilibrium conditions, the conductance decreases under lower biases and fluctuates as the bias increases, and it is reduced for almost all devices under voltages. However, the amplitude of the variations differs. The best doping for enhancing conductance can reach 37%, mainly at low voltages, while the most effective attenuation appears in the Mg-small doping, with a variation rate of −23%. In contrast, the current increases linearly with the bias for all devices. The current of the new-type device can achieve growth (up to 31%) at low voltages and reduction (up to 16%) at high voltages via the doping of metal elements. These results help us to understand the effects of defects on the electronic properties of new 2D iodine materials and provide a solid support for the applications of new 2D iodine materials in controllable electronic devices and sensors.

## Figures and Tables

**Table 1:** Changes in and variation rates of equilibrium electron transport properties for doped new 2D iodine-based devices compared to the undoped 2D iodine-based device. Here, Con, TS, and DDOS are short for equilibrium conductance, electron transmission possibility at the Fermi level (E_F_), and device density of states at E_F_, respectively.

Dopants	Doping Site	Changes in Con (G0)	Variation Rate (%)	Changes in TS	Variation Rate (%)	Changes in DDOS (eV^−1^)	Variation Rate (%)
Na	big	0.2874	13.36%	0.4623	23.01%	4.6973	24.15%
	small	0.4903	22.78%	0.6594	32.82%	4.8661	25.01%
K	big	0.5877	27.31%	0.6279	31.25%	4.8935	25.15%
	small	0.3463	16.09%	0.5428	27.02%	6.4726	33.27%
Mg	big	0.1417	6.58%	0.1293	6.44%	3.7493	19.27%
	small	0.0775	3.60%	0.0849	4.22%	2.5120	12.91%
Ca	big	0.2442	11.35%	0.4250	21.15%	10.9708	56.39%
	small	0.4883	22.69%	0.5842	29.08%	2.8705	14.76%
Al	big	0.6207	28.84%	0.6949	34.59%	6.4266	33.04%
	small	0.3648	16.95%	0.4781	23.80%	4.9305	25.35%
Ga	big	0.1880	8.73%	0.2142	10.66%	3.9206	20.15%
	small	0.1637	7.61%	0.1835	9.14%	0.1126	0.58%

## Data Availability

Data will be made available on request.

## References

[1] Glavin N.R., Rao R., Varshney V., Bianco E., Apte A., Roy A., Ringe E., Ajayan P.M. (2020). 2D Materials: Emerging Applications of Elemental 2D Materials (Adv. Mater. 7/2020). Adv. Mater..

[2] Liu Y., Huang Y., Duan X. (2019). Van der Waals integration before and beyond two-dimensional materials. Nature.

[3] Li M., Kang J.S., Nguyen H.D., Wu H., Aoki T., Hu Y. (2019). Anisotropic Thermal Boundary Resistance across 2D Black Phosphorus: Experiment and Atomistic Modeling of Interfacial Energy Transport. Adv. Mater..

[4] Kim S., Cui J., Dravid V.P., He K. (2019). Orientation-Dependent Intercalation Channels for Lithium and Sodium in Black Phosphorus. Adv. Mater..

[5] Novoselov K.S., Fal′ko V.I., Colombo L., Gellert P.R., Schwab M.G., Kim K. (2012). A roadmap for graphene. Nature.

[6] Novoselov K.S., Andreeva D.V., Ren W., Shan G. (2019). Graphene and other two-dimensional materials. Front. Phys..

[7] Molle A., Goldberger J., Houssa M., Xu Y., Zhang S.-C., Akinwande D. (2017). Buckled two-dimensional Xene sheets. Nat. Mater..

[8] Tan C., Cao X., Wu X.-J., He Q., Yang J., Zhang X., Chen J., Zhao W., Han S., Nam G.-H. (2017). Recent Advances in Ultrathin Two-Dimensional Nanomaterials. Chem. Rev..

[9] Mannix A.J., Zhou X.-F., Kiraly B., Wood J.D., Alducin D., Myers B.D., Liu X., Fisher B.L., Santiago U., Guest J.R. (2015). Synthesis of borophenes: Anisotropic, two-dimensional boron polymorphs. Science.

[10] Carvalho A., Wang M., Zhu X., Rodin A.S., Su H., Castro Neto A.H. (2016). Phosphorene: From theory to applications. Nat. Rev. Mater..

[11] Hu Y., Liang J., Xia Y., Zhao C., Jiang M., Ma J., Tie Z., Jin Z. (2022). 2D Arsenene and Arsenic Materials: Fundamental Properties, Preparation, and Applications. Small.

[12] Niu T., Meng Q., Zhou D., Si N., Zhai S., Hao X., Zhou M., Fuchs H. (2020). Large-Scale Synthesis of Strain-Tunable Semiconducting Antimonene on Copper Oxide. Adv. Mater..

[13] Reis F., Li G., Dudy L., Bauernfeind M., Glass S., Hanke W., Thomale R., Schäfer J., Claessen R. (2017). Bismuthene on a SiC substrate: A candidate for a high-temperature quantum spin Hall material. Science.

[14] Qin J., Qiu G., Jian J., Zhou H., Yang L., Charnas A., Zemlyanov D.Y., Xu C.-Y., Xu X., Wu W. (2017). Controlled Growth of a Large-Size 2D Selenium Nanosheet and Its Electronic and Optoelectronic Applications. ACS Nano.

[15] Wang Y., Qiu G., Wang R., Huang S., Wang Q., Liu Y., Du Y., Goddard W.A., Kim M.J., Xu X. (2018). Field-effect transistors made from solution-grown two-dimensional tellurene. Nat. Electron..

[16] Xia F., Wang H., Jia Y. (2014). Rediscovering black phosphorus as an anisotropic layered material for optoelectronics and electronics. Nat. Commun..

[17] Cui S., Pu H., Wells S.A., Wen Z., Mao S., Chang J., Hersam M.C., Chen J. (2015). Ultrahigh sensitivity and layer-dependent sensing performance of phosphorene-based gas sensors. Nat. Commun..

[18] Zhou J., Chen J., Chen M., Wang J., Liu X., Wei B., Wang Z., Li J., Gu L., Zhang Q. (2019). Few-Layer Bismuthene with Anisotropic Expansion for High-Areal-Capacity Sodium-Ion Batteries. Adv. Mater..

[19] Chimene D., Alge D.L., Gaharwar A.K. (2015). Two-Dimensional Nanomaterials for Biomedical Applications: Emerging Trends and Future Prospects. Adv. Mater..

[20] Gusmão R., Sofer Z., Bouša D., Pumera M. (2017). Pnictogen (As, Sb, Bi) Nanosheets for Electrochemical Applications Are Produced by Shear Exfoliation Using Kitchen Blenders. Angew. Chem. Int. Edit..

[21] Wei Q., Chen J., Ding P., Shen B., Yin J., Xu F., Xia Y., Liu Z. (2018). Synthesis of Easily Transferred 2D Layered BiI_3_ Nanoplates for Flexible Visible-Light Photodetectors. ACS Appl. Mater. Interfaces.

[22] Zhong M., Huang L., Deng H.-X., Wang X., Li B., Wei Z., Li J. (2016). Flexible photodetectors based on phase dependent PbI_2_ single crystals. J. Mater. Chem. C.

[23] Wang F., Zhang Z., Zhang Y., Nie A., Zhao W., Wang D., Huang F., Zhai T. (2020). Honeycomb RhI_3_ Flakes with High Environmental Stability for Optoelectronics. Adv. Mater..

[24] Hulkko E., Kiljunen T., Kiviniemi T., Pettersson M. (2009). From Monomer to Bulk: Appearance of the Structural Motif of Solid Iodine in Small Clusters. J. Am. Chem. Soc..

[25] Qian M., Xu Z., Wang Z., Wei B., Wang H., Hu S., Liu L.M., Guo L. (2020). Realizing Few-Layer Iodinene for High-Rate Sodium-Ion Batteries. Adv. Mater..

[26] Huran A.W., Wang H.-C., San-Miguel A., Marques M.A.L. (2021). Atomically Thin Pythagorean Tilings in Two Dimensions. J. Phys. Chem. Lett..

[27] Liu P., Zhang G., Yan Y., Jia G., Liu C., Wang B., Yin H. (2021). Strain-tunable phase transition and doping-induced magnetism in iodinene. Appl. Phys. Lett..

[28] Bafekry A., Stampfl C., Faraji M., Mortazavi B., Fadlallah M.M., Nguyen C.V., Fazeli S., Ghergherehchi M. (2022). Monoelemental two-dimensional iodinene nanosheets: A first-principles study of the electronic and optical properties. J. Phys. D Appl. Phys..

[29] Kuang C., Zeng W., Qian M., Liu X. (2021). Liquid-Phase Exfoliated Few-Layer Iodine Nanosheets for High-Rate Lithium-Iodine Batteries. ChemPlusChem.

[30] You Y., Zhu Y.-X., Jiang J., Chen Z., Wu C., Zhang Z., Lin H., Shi J. (2023). Iodinene Nanosheet-to-Iodine Molecule Allotropic Transformation for Antibiosis. J. Am. Chem. Soc..

[31] Liu H., Cao S., Chen L., Zhao K., Wang C., Li M., Shen S., Wang W., Ge L. (2022). Electron acceptor design for 2D/2D iodinene/carbon nitride heterojunction boosting charge transfer and CO_2_ photoreduction. Chem. Eng. J..

[32] Wen T., Wang Y., Li N., Zhang Q., Zhao Y., Yang W., Zhao Y., Mao H.-k. (2019). Pressure-Driven Reversible Switching between n- and p-Type Conduction in Chalcopyrite CuFeS_2_. J. Am. Chem. Soc..

[33] Bu K., Luo H., Guo S., Li M., Wang D., Dong H., Ding Y., Yang W., Lü X. (2020). Pressure-Regulated Dynamic Stereochemical Role of Lone-Pair Electrons in Layered Bi_2_O_2_S. J. Phys. Chem. Lett..

[34] Zhang Q., Liu X., Li N., Wang B., Huang Q., Wang L., Zhang D., Wang Y., Yang W. (2020). Pressure-driven chemical lock-in structure and optical properties in Sillen compounds PbBiO_2_X (X = Cl, Br, and I). J. Mater. Chem. A.

[35] Duan D., Jin X., Ma Y., Cui T., Liu B., Zou G. (2009). Effect of nonhydrostatic pressure on superconductivity of monatomic iodine: An ab initio study. Phys. Rev. B.

[36] Sakai N., Takemura K.-i., Tsuji K. (1982). Electrical properties of high-pressure metallic modification of iodine. J. Phys. Soc. Japan.

[37] Kenichi T., Kyoko S., Hiroshi F., Mitsuko O. (2003). Modulated structure of solid iodine during its molecular dissociation under high pressure. Nature.

[38] San Miguel A., Libotte H., Gaspard J.P., Gauthier M., Itié J.P., Polian A. (2000). Bromine metallization studied by X-ray absorption spectroscopy. Eur. Phys. J. B Condens. Matter Complex Syst..

[39] Li Z., Li H., Liu N., Du M., Jin X., Li Q., Du Y., Guo L., Liu B. (2021). Pressure Engineering for Extending Spectral Response Range and Enhancing Photoelectric Properties of Iodine. Adv. Opt. Mater..

[40] Song D., Liu K., Li J., Zhu H., Sun L., Okulov A. (2023). Mechanical tensile behavior-induced multi-level electronic transport of ultra-thin SiC NWs. Mater. Today Commun..

[41] Liu K., Li Y., Liu Q., Song D., Xie X., Duan Y., Wang Y., Li J. (2022). Superior electron transport of ultra-thin SiC nanowires with one impending tensile monatomic chain. Vacuum.

[42] Mlinar V. (2017). Electronic and optical properties of nanostructured MoS_2_ materials: Influence of reduced spatial dimensions and edge effects. Phys. Chem. Chem. Phys..

[43] Liu K., Li J., Liu R., Li H., Okulov A. (2023). Non-equilibrium electronic properties of ultra-thin SiC NWs influenced by the tensile strain. J. Mater. Res. Technol..

[44] Li J., Li T., Zhou Y., Wu W., Zhang L., Li H. (2016). Distinctive electron transport on pyridine-linked molecular junctions with narrow monolayer graphene nanoribbon electrodes compared with metal electrodes and graphene electrodes. Phys. Chem. Chem. Phys..

[45] Cao J., Dong J., Saglik K., Zhang D., Solco S.F.D., You I.J.W.J., Liu H., Zhu Q., Xu J., Wu J. (2023). Non-equilibrium strategy for enhancing thermoelectric properties and improving stability of AgSbTe_2_. Nano Energy.

[46] Brandbyge M., Mozos J.-L., Ordejón P., Taylor J., Stokbro K. (2002). Density-functional method for nonequilibrium electron transport. Phys. Rev. B.

[47] Taylor J., Guo H., Wang J. (2001). Ab initio modeling of quantum transport properties of molecular electronic devices. Phys. Rev. B.

[48] Zhang L., Yuan J., Shen L., Fletcher C., Li H. (2019). Taper-shaped carbon-based spin filter. Appl. Surf. Sci..

[49] Landauer R. (1970). Electrical resistance of disordered one-dimensional lattices. Philos. Mag..

[50] Reed M.A., Zhou C., Muller C., Burgin T., Tour J. (1997). Conductance of a molecular junction. Science.

[51] Datta S. (1997). Electronic Transport in Mesoscopic Systems.

